# First proof-of-concept evaluation of the FUSION-X-US-II prototype for the performance of automated breast ultrasound in healthy volunteers

**DOI:** 10.1007/s00404-021-06081-z

**Published:** 2021-05-10

**Authors:** Benedikt Schaefgen, Marija Juskic, Madeleine Hertel, Richard G. Barr, Marcus Radicke, Anne Stieber, André Hennigs, Fabian Riedel, Christof Sohn, Joerg Heil, Michael Golatta

**Affiliations:** 1 Department of Gynecology and Obstetrics, University Breast Unit, Heidelberg, Germany; 2 Northeastern Ohio Medical University and Southwoods Imaging, Youngstown, OH USA; 3 Siemens Healthcare GmbH, Forchheim, Germany; 4 Department of Radiology, University Breast Unit, Heidelberg, Germany

**Keywords:** Breast cancer, Diagnostics, Automated breast ultrasound, Digital breast tomosynthesis, Multimodal imaging

## Abstract

**Purpose:**

The FUSION-X-US-II prototype was developed to combine 3D-automated breast ultrasound (ABUS) and digital breast tomosynthesis in a single device without decompressing the breast. We evaluated the technical function, feasibility of the examination workflow, image quality, breast tissue coverage and patient comfort of the ABUS device of the new prototype.

**Methods:**

In this prospective feasibility study, the FUSION-X-US-II prototype was used to perform ABUS in 30 healthy volunteers without history of breast cancer. The ABUS images of the prototype were interpreted by a physician with specialization in breast diagnostics. Any detected lesions were measured and classified using BI-RADS^®^ scores. Image quality was rated subjectively by the physician and coverage of the breast was measured. Patient comfort was evaluated by a questionnaire after the examination.

**Results:**

One hundred and six scans were performed (61 × CC, 23 × ML, 22 × MLO) in 60 breasts. Image acquisition and processing by the prototype was fast and accurate. Breast coverage by ABUS was approximately 90.8%. Sixteen breast lesions (all benign, classified as BIRADS^®^ 2) were identified. The examination was tolerated by all patients.

**Conclusion:**

The FUSION-X-US-II prototype allows a rapid ABUS scan with mostly high patient comfort. Technical developments resulted in an improvement of quality and coverage compared to previous prototype versions. The results are encouraging for a test of the prototype in a clinical setting in combination with tomosynthesis.

## Introduction

Mammography is the main pillar of breast cancer screening with the goal of early diagnosis and treatment. Dense breast tissue leads to a decrease in sensitivity down to 48% [1] and is also an independent risk factor for developing breast cancer. In this group with elevated risk, hand-held ultrasound (HHUS) is able to detect additional malignancies but it is time consuming and examiner dependent [1–3].

Both, mammography and HHUS have undergone further development addressing these shortcomings. One is digital breast tomosynthesis, a 3D procedure which increases sensitivity and specificity by reducing tissue overlapping, the main cause of false positive and false negative findings in 2D-mammography [4–6].

3D-automated breast ultrasound (ABUS) has been developed to overcome the shortcomings of HHUS. It offers higher interobserver reliability and is a time-saving procedure [7–9]. Several studies have shown that additional ABUS to mammography can increase the cancer detection rate in a screening situation [8–11].

To combine mammography/tomosynthesis and ABUS various systems with different approaches to scan the whole breast have been designed. All studies showed the possibility of ABUS to detect most of the lesions seen in mammography/tomosynthesis. Padilla et al. were able to detect one additional cancer by adding ABUS to tomosynthesis (sensitivity 96% with tomosynthesis vs. 100% with tomosynthesis + ABUS). Both image quality and coverage showed limitations with the need for technical improvement [12–17].

Previously, we evaluated the FUSION-X-US prototype (Siemens Healthcare GmbH, Forchheim, Germany) which allows ABUS to be performed after tomosynthesis, while the breast is still under compression without a change in the position of the breast. This device offered a technically reliable, promising method for breast diagnostics; however, image quality and breast coverage of ABUS was limited [13]. Based on the previous prototype, the FUSION-X-US-II prototype has been further developed with several technical advancements.

In this proof-of-concept study, we assessed the technical feasibility of performing ABUS in healthy volunteers with the new prototype. Secondary endpoints were image quality, coverage of the breast and patient comfort.

## Materials and methods

The study protocol was approved by the local ethics committee (Medical Faculty Heidelberg, reference number S-438/2018) and consistent with the Health Information Portability and Accountability Act of 1996 (HIPAA), with written informed consent of every participant enrolled in the study.

### Equipment

The FUSION-X-US-II consists of the ACUSON S2000 automated breast volume scanner (ABVS, Siemens Healthcare GmbH, Mountain View, CA, USA) and the MAMMOMAT Inspiration (Siemens Healthcare GmbH, Forchheim, Germany), both FDA approved and CE certified and used in routine clinical practice. The prototype combining both techniques is a research device and is not commercially available. So far, the prototype cannot be used to perform targeted biopsies.

**Fig.1:**
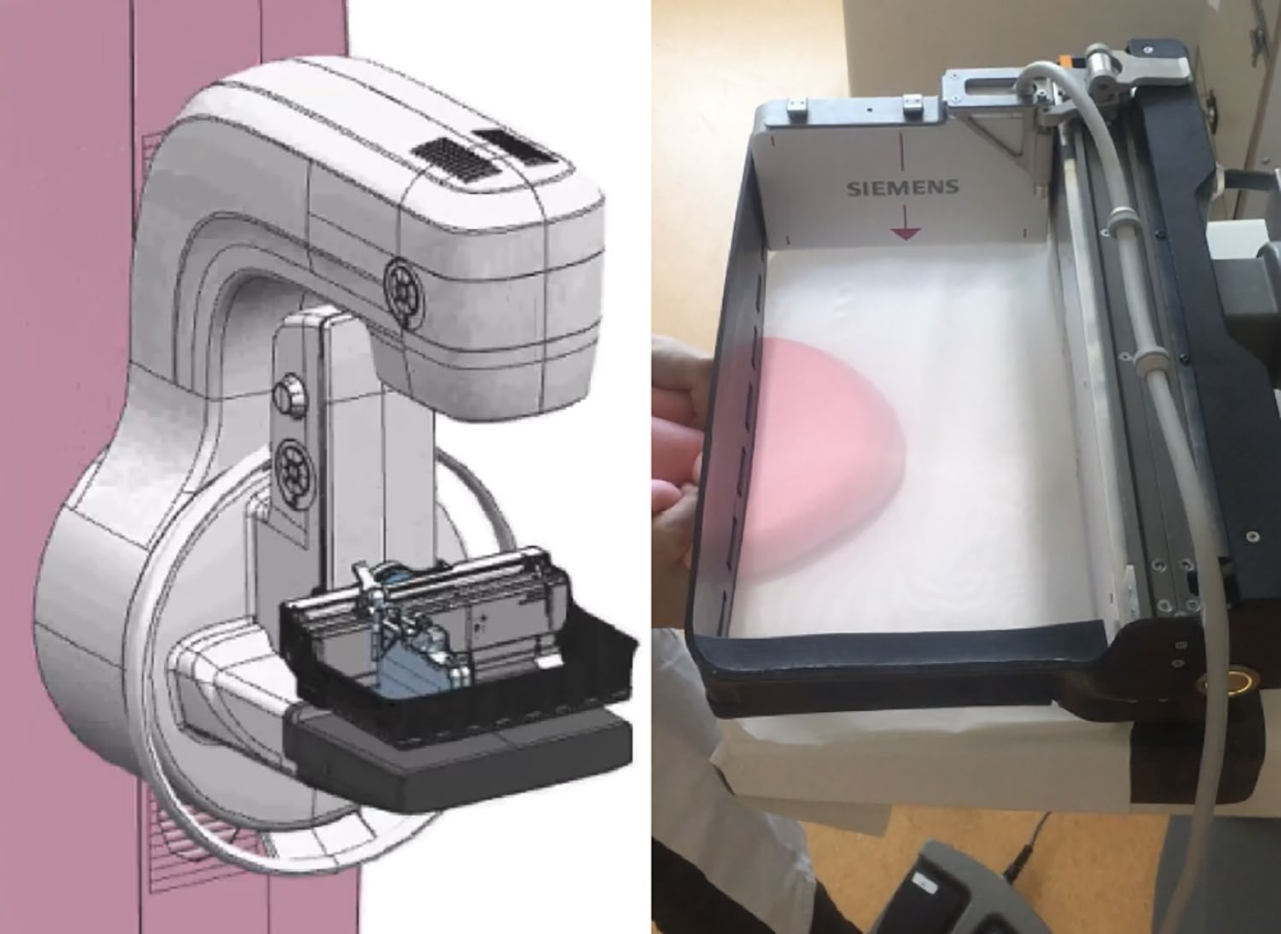
**a** **Schematic view of the FUSION-X-US-II prototype.** A specially developed compression paddle, which is composed of a rigid frame and a gauze, is inserted in a standard MAMMOMAT Inspiration. The ultrasound transducer is included in the prototype compression paddle. **b** **Breast phantom under compression.** The FUSION-X-US-II prototype as build up for the study is used to simulate the breast compression with a breast phantom by using the prototype compression paddle and the additional air cushion

For this study evaluating the performance of ABUS in healthy volunteers, only the ACUSON S2000 ABVS device was used and the participants were not exposed to any radiation. The 5–14 MHz linear ultrasound transducer with an array length of 154 mm is integrated into the compression paddle, so the ABUS can be performed under the same compression and position as tomosynthesis. A specially developed compression paddle was used, which is composed of a rigid frame and specially woven gauze being radiolucent and permeable for ultrasound lotion. The gauze is able to sustain forces adequate for performing mammography/tomosynthesis and ABUS of over 200 N. In comparison to the previous FUSION-X-US, the paddle was adapted in terms of weight and size to provide better positioning and an improved tightening mechanism of the gauze enables a more conform compression. The transducer system is connected to the ACUSON S2000 ABVS device, where the acquired scans are displayed and saved. To further improve the contact between the breast surface and the ultrasound probe in FUSION-X-US-II, a special air cushion has been designed, which is inflated after lowering the compression paddle and adds homogeneous pressure from caudal, pushing the peripheral parts of the breast towards the gauze (Fig. 1). The inflation is controlled manually by the radiology technologist until optimal contact with the gauze is reached.

The breast is positioned in a standard view [craniocaudal (CC), mediolateral-oblique (MLO) or mediolateral (ML)] and the compression paddle is lowered. The air cushion is inflated and coupling lotion is applied on the gauze either manually or through an integrated automatic dispensing device. The transducer moves automatically over the breast, covering an area of maximum 30 × 15 cm^2^ with a maximum penetration depth of 10 cm. When the final position of the probe is reached, the gauze is lifted to release the breast.

The data are transferred to the working station and processed to generate images for interpretation by the physician.

### Study design

This monocentric, prospective study was performed in September 2018 with 30 healthy women volunteering to participate. We included women aged 18 or older, non-pregnant, capable of understanding the study constraints after written informed consent. All participants received ABUS using the prototype, without, however, conducting tomosynthesis or mammography; so no radiation was applied.

The breast was positioned under the compression paddle by a radiology technician. The force and the breast thickness during compression were documented for each position. Before the first scan was started, the ultrasound probe was positioned manually on the breast for real-time adjustment of frequency, focal depth and gain at the discretion of the investigator. Each breast was scanned in CC projection and additional scans in MLO or ML (projection) were obtained. CC orientation was defined as the standard view of ABUS. In cases of excellent patient’s tolerability, we decided to perform an additional scan in a second orientation (ML/MLO). The intention was to test if also the other orientations could be scanned using the prototype, because this is necessary if the prototype is used to obtain ABUS in combination with tomosynthesis (which will often be performed in ML/MLO). We aimed at an equal distribution of ML and MLO scans.

ABUS images were evaluated by an experienced physician with over 10 years of experience with ABUS systems using the syngo.breast ultrasound software (Software Version VA25, ©2012-2013 Siemens Medical Solutions USA, Inc., PA, USA). According to the ACR guidelines, the breast density was assessed in three categories (homogeneous fatty background texture, homogeneous fibroglandular background texture, heterogeneous background texture). Image quality was rated subjectively by the physician on a scale ranged from 1 to 5. Category 1 represents a very low quality, with no identifiable breast structures; category 2, a quality below diagnostic applicability with identifiable breast structures; category 3, a sufficient quality for diagnostic applicability (lower than HHUS); category 4, a quality close to/comparable to HHUS; and category 5, an equal or higher quality compared to HHUS.

All detected lesions were measured and classified using BI-RADS^®^ scores [18].

**Fig. 2:**
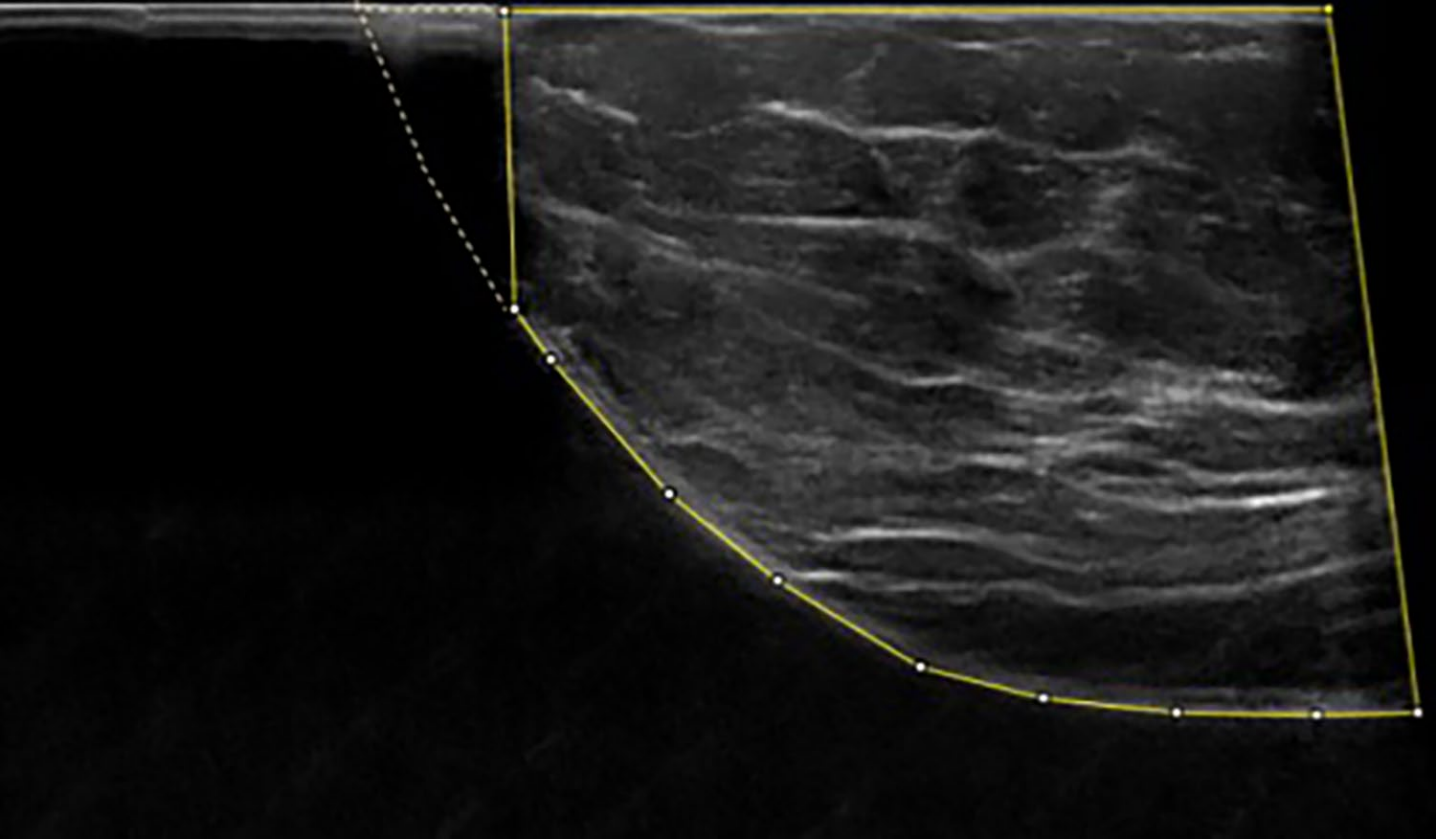
**Extrapolation of the breast coverage.** The breast area covered is calculated with a polygon tool (solid line). The estimated total breast area is determined through manual extrapolation along the skin assuming a continuous skin contour (dotted outline)

To quantify the breast coverage by the ABUS, the level of the nipple region in the US image was used as a reference of comparison. The breast area depicted in this ABUS image (solid outline in Fig. 2) was measured using the software Fiji (ImageJ, Version 2.0.0, 2018, ©2010–2020). The estimated total breast area was determined through manual extrapolation along the skin (dotted outline in Fig. 2), assuming a continuous skin contour. The quotient of the measured breast area (covered by ABUS) and the extrapolated total breast area was used to estimate the percentage of the breast area covered in ABUS.

After the scan, the participants were asked by a study assistant to rate (a) the tolerability of the breast compression and (b) their perception of the additional force applied by the inflated air cushion from 1 (comfortable) to 5 (very uncomfortable).

### Statistical analysis

This is an explorative study based on descriptive statistics. Values are given as mean with standard deviation or median with quartiles dependent on the level of measurement. The differences in quality over the projections were tested with Fisher’s exact test. The resulting *p* value has to be interpreted descriptively.

## Results

The combination of tomosynthesis and ABUS in one workflow aims to combine the advantages of both imaging techniques and provide precise spatial correlation of mammographic and ultrasound findings.

### Study participants

Thirty participants aged from 20 to 60 years (mean: 28.2, SD: 10.4) were included. A total of 106 scans were performed (61 scans in CC, 23 in ML and 22 in MLO projection) (see Table 1).

**Table 1:** Cohort description

Sample size^a^	30
Age^b^	28.2 (10.4)
Number of ABUS^a^	106
CC	61 (57.5)
ML	23 (21.7)
MLO	22 (20.8)
Force used for compression(N)^b^	84.6 (24.1)
Breast thickness under compression (mm)^b^	45.8 (13.9)

^a^Values are absolute frequencies. Relative frequencies are given as percentages in parentheses. Percentages are rounded

^b^Values are means with standard deviation in parentheses

The ABUS device was technically reliably and all attempted scans were successfully completed. Each scan took between 40 and 60 s depending on the breast thickness. 29 participants were scanned in standing position, one participant was scanned in sitting position because of minor circulatory problems, which had otherwise no effect on the workflow.

The data processing of the DICOM files to the interpretation work station was correct and a complete set of images was digitally reconstructed for all cases.

**Fig. 3:**
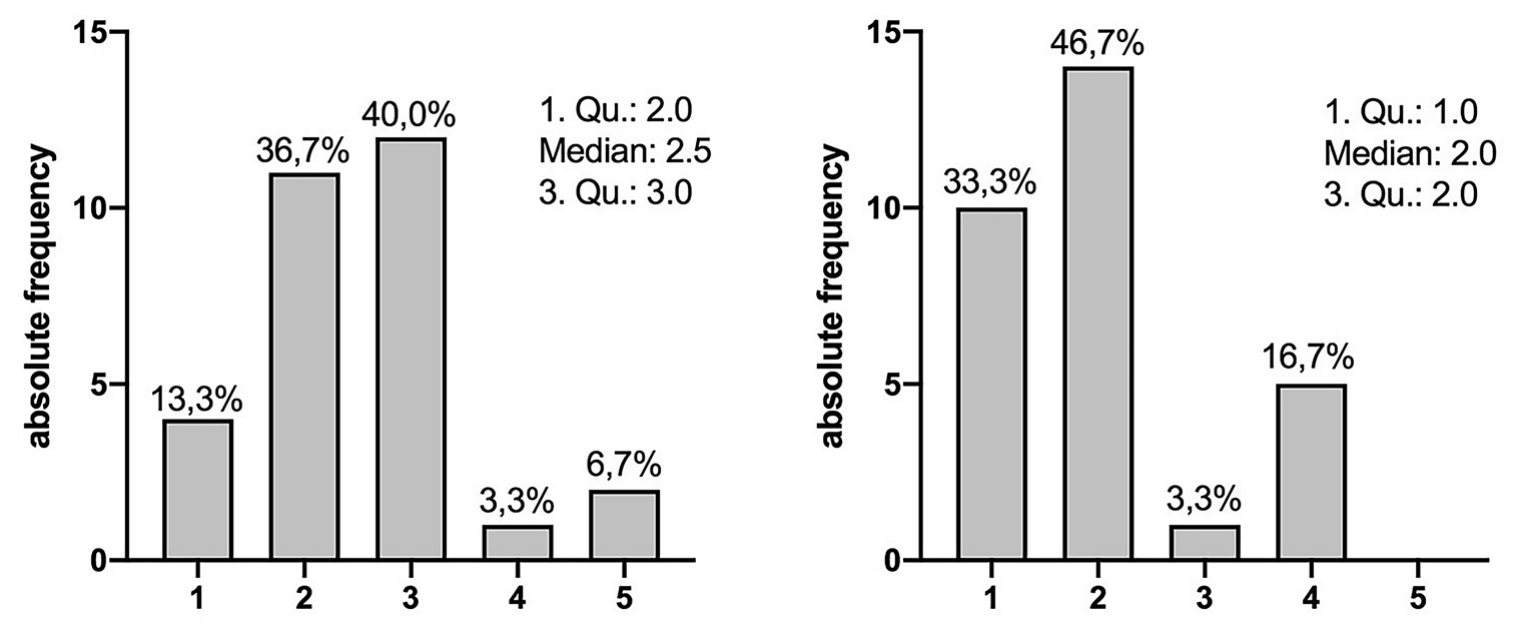
**a** Patient comfort during breast compression and examination. Tolerability was rated on a scale from 1 (very good tolerability) to 5 (bad tolerability). **b** Change of the patient comfort with homogenized pressure distribution through use of the air cushion rated on a scale from 1 (much better than without the cushion) to 5 (much worse than without the cushion)

The completion of all scans was well tolerated by the participants. Retrospectively, 90.0% of the participants rated tolerability as very good to moderate (1–3), 3.3% as uncomfortable and 6.7% as very uncomfortable (Fig. 3a). The inflation of the air cushion generally had a positive effect on patient comfort. 46.7% of patients reported that tolerability of the breast compression got better after inflation of the air cushion, 33.3% of patients said that tolerability got much better with the cushion. Only 16.7% of patients reported a slight decrease in comfort due to the air cushion (Fig. 3b).

### Interpretation of the ABUS images

To evaluate the breast area covered by the ABUS, the acquired US image was extrapolated as shown in Fig. 1. Thereby, the area covered by the ABUS was on average 25.69 cm^2^ (ranging from 0.69 to 66.21 cm^2^), while the area towards the nipple not covered averaged on 2.56 cm^2^ (ranging from 0 to 34.66 cm^2^). In total, 90.8% (ranging from 30.0 to 100.0%) of the estimated (extrapolated) breast area was covered by the ABUS scan.

Echotexture of the breast was described as homogeneous fatty (2/60, 3.3%), homogeneous fibroglandular (23/60, 38.3%) and heterogeneous (35/60, 58.3%).

In 16 of 60 breasts (26.7%), a breast lesion was detected. The size of the lesions was on average 5.7 mm, ranging from 2.0 to 20.0 mm. All lesions were classified as benign (BIRADS^®^ 2). The typical sonographic features of cysts or duct ectasia were clearly identifiable in the ABUS image (see Table 2).

**Table 2:** Breast ultrasound results

Background echotexture^a^	
Homogeneous fatty	2 (3.3)
Homogeneous fibroglandular	23 (38.3)
Heterogeneous	35 (58.3)
Image quality^a^	
1	2 (1.9)
2	20 (18.9)
3	60 (56.6)
4	24 (22.6)
5	0 (0.0)
Cases with notable artifacts^a^	61 (57.7)
Artifact area (cm^2^)^b^	3.52 (0.10–9.90)
Lesions described^a^	16 (26.7)
Size (mm)^b^	5.7 (2.0–20.0)
Coverage	
Extrapolated total breast area (cm^2^)^b^	25.27 (0.69–71.19)
Covered breast area (cm^2^)^b^	21.43 (0.69–66.21)
Percentage of total area (%)^b^	90.8 (30.0–100.0)

^a^The values are absolute frequencies. The numbers in parentheses are percentages. The percentages are rounded

^b^The values are means or medians dependent on the level of measurement. The minimum and maximum value is given in parentheses

Image quality was rated on a scale from 1 (poor quality, tissue structures indistinguishable) to 5 (quality superior to HHUS) with a mean quality of 3 (lower quality compared to HHUS, but well distinguishable tissue structures). 24 of the 106 scans (22.6%) were rated to be of quality comparable to HHUS. None of the scans was rated as 5 (equal or higher quality than HHUS). In two cases, the quality was rated as 1, as no tissue structures were identifiable. There was no significant difference in the rating of image quality over the three projections (*p* = 0.70, see Table 3).

**Table 3:** Image quality by projection

	Projection			*p* value^*^
	CC	MLO	ML	
Quality				
1	1 (1.7)	0 (0.0)	1 (4.6)	0.70
2	11 (18.0)	6 (26.1)	3 (13.6)	
3	33 (54.1)	14 (60.9)	13 (59.1)	
4	16 (26.2)	3 (13.0)	5 (22.7)	
5	0 (0.0)	0 (0.0)	0 (0.0)	
Total	61	23	22	

The values are absolute frequencies. Numbers in parentheses are column percentages. Percentages are rounded

^*^The *p* value is based on the Fishers exact test

In 61 out of 106 cases (57.5%), ultrasound artifacts obscuring a part of the breast tissue with an average area of 3.52 cm^2^ (ranging from 0.10 to 9.90 cm^2^) were identified by the physician.

## Discussion

The FUSION-X-US-II prototype was designed to address the limitations of the preceding prototype [13], most importantly breast coverage of ABUS. The principal goal of this proof-of-concept study was to evaluate the technical reliability of the prototype and the feasibility of the diagnostic workflow. Secondly, ABUS coverage and image quality as well as patients’ tolerability of the exam were assessed. The FUSION-X-US-II prototype worked technically reliable allowing all study participants to be scanned in standard projections.

### Image quality

Overall, in 79.2% of the cases, ABUS quality was rated category 3 or higher, which means that tissue structures were clearly visible and ABUS images were of diagnostic use. Moreover, quality was rated as 4/5 in 22.6% of cases. Generally, image quality deteriorated with increasing penetration depth. This phenomenon was more pronounced in larger breasts, where caudal parts of the breast presented in lower contrast and quality. The lack of image quality can be explained by the technical specifications of the transducer used in this study, which has already been introduced in 2008 [19] and was designed to be used in supine position rather than in CC, ML or MLO position. The challenge of providing good image quality within a sufficient penetration range could only be met with current high-end transducers that benefit from recent technical advancements in hardware and software. It should be emphasized that the concept of automated scanning is not the reason for current limitation of image quality. Adapting a state-of-the-art breast transducer to fit the requirements of FUSION-XUS-II prototype seems to be a crucial step to achieve reliable image quality.

### Clinical workflow

The prototype device was designed to provide a fast, semiautomatic and thorough scanning of the breast, which could potentially be used in a screening situation to detect lesions requiring further diagnostic workup. As long as there is a significant difference in image quality between HHUS and ABUS, HHUS will remain indispensable to further investigate and classify any detected lesions with high resolution. On the other hand, the implication of automated scanning devices has several advantages for the clinical workflow, e.g., a higher grade of standardization and better comparability with prior images. Additionally, the ABUS workflow allows the physician to evaluate the images at any time after the scan has been performed by the radiology technician, which can be an important time- and cost-effective benefit in a clinical setting. Still, ABUS has some limitations in comparison to HHUS, and it can only supplement but not replace HHUS in the clinical workflow. The examination of the axilla, which is an important part of a thorough sonographic examination, cannot be assessed using ABUS. Furthermore, ABUS allows no direct confirmation of any suspicious findings the same examination (unlike HHUS, which can be used to guide a core cut biopsy immediately). Another important aspect is that the automatization of the ultrasound examination reduces the personal contact between patient and physician and the results cannot be demonstrated to and discussed directly with the patient.

### Coverage

The estimation of the breast coverage is difficult, since only ABUS was performed and there is no objective 3D assessment of the breast as a gold standard. The coverage calculated in this study on the basis of an extrapolated 2D area based on the ABUS images is only an approximation. Still, ABUS covered the largest part of the breast in most of the cases. Only in two cases a coverage lower than 50% was achieved. Both scans were performed in the same patient sensitive to pain resulting in positioning problems and no sufficient coupling of the breast and ultrasound traducer, so that only a part of the breast close to the thoracic wall was visible in ABUS. However, the average area not covered by ABUS of 2.56 cm^2^ (9.2% of the extrapolated total breast area) means that a thorough examination of the whole breast with the ABUS alone is not possible with the prototype.

One reason for insufficient coverage is that the breast does not have contact at all sides with the gauze (and therefore the ultrasound probe) due to its geometric shape. Even under compression, the most ventral part of the breast is convex and does not have full contact with the gauze. The use of an air cushion to ease compression and lift up the outer parts of the breast helped to increase the coverage but should be optimized to equally support all breast shapes.

Additionally, the ABUS transducer is embedded into a static metal housing with a thickness of 1 cm, which leads to a gap between the active area of the transducer and the thoracic wall. Clinically, this limitation can be relevant as lesions that are located in this area might not be detected by ABUS. Further investigations to adapt the transducer to meet this technical challenge are needed. In this study, the exact area near the thoracic wall, which was not covered by the scan, could not be assessed. In future studies, the error will be quantified by comparing the breast volume scanned in ABUS to the breast volume in tomosynthesis. Current ABUS devices cannot be used to examine the axilla, and so this region is also inaccessible with the prototype.

### Artifacts

Notable artifacts were described in 57.5% of the cases. Streaky artifacts appeared when the gauze was not sufficiently covered with ultrasound coupling lotion. We dispensed ultrasound lotion on the gauze, but in some areas, the lotion was pushed aside by the ultrasound probe, resulting in a loss of ultrasound contact in these areas. Round artifacts emerged mostly on the ventral (close to the nipple) and dorsal edge (close to the chest wall) because contact with the gauze and the transducer in these areas was poorer than in the center due to the geometric shape of the breast.

### Breast lesions

We did not detect any suspicious lesions, as could be expected in a study population of young, healthy volunteers. Nevertheless, we described several benign cysts with a minimum size of 2.0 × 2.0 mm^2^. This can be interpreted as a proof of concept that the prototype can be used to differentiate breast parenchyma from breast lesions. The possibility to diagnose breast lesions was not an endpoint of this study and has to be evaluated in a larger study.

### Patient comfort

Patient comfort was generally good with only 9.9% of the patients reporting an uncomfortable or very uncomfortable experience. In comparison with studies reporting the tolerability of mammography (in which similar compression is applied), this is a positive result [20]. It should be kept in mind that the large majority of study participants have never had a mammography before, so they experienced this form of breast compression for the first time. The inflation of the air cushion had a positive effect on patient comfort in 80.0% of the cases.

## Conclusion

The FUSION X-US-II prototype allows ABUS scans of compressed breasts to be performed semi-automatically in a swift workflow in the standard orientations. The largest part of the breast area can be covered with sufficient image quality, but further improvements are necessary for routine clinical use. The combination of ABUS and tomosynthesis through the prototype will be tested in a prospective study in a clinical setting.

## Data Availability

Data and material can be made available upon request.
